# Taxation without Representation

**Published:** 1888-03

**Authors:** R. P. Talley

**Affiliations:** Belton, Texas


### "TAXATION WITHOUT REPRESENTATION."

LETTER FROM DR. TALLEY.

BELTON, TEXAS, February 10, 1888.

*Editor Daniel's Texas Medical Journal:*

In your article in Vol. III, No. 7, page 291, "Taxation without Representation," I think you very unjustly criticize the constitution and by-laws of the Texas State Medical Associatlon. You refer to one of the clauses as "iniquitous," when in fact an analysis of the whole subject shows, to my mind, that this hard adjective of yours to-wit: "iniquitous," had better be applied to the *workings* of the Association. You say: "our plan of electing officers, in particular, is unfair, the *majority* are not represented in the choice of officers, yet, at the same time, they are taxed." Now, as is well known, the nominating committee has always elected the one nominated by a *majority* vote. The committee then reports as "the law directs." The report is then before and received by the Association for just such action as the Association may see cause to adopt. Any member of the Association in or out of the nominating committee can object by amendments etc., to any part or all of the report and thus bring a majority, and in many cases, if managed in a proper parlimentary way, a two-third vote, to bear upon any important question thus involved. The real trouble comes thusly, to-wit: There is always *all sort* of wrangling for office, etc., for *self* or *friends*, and by this the Association, for the time being, is in a sort of mob meeting, and when the committee reports some member without any great regard or respect for any parlimentary law, moves that the report be adopted, and some one or more second the motion. The presiding officer puts the question, upon which a few members vote yes, and to save trouble, etc., no one offers to vote no; while perhaps more than the majority of the members then present are engaged in talking on all sorts of questions, etc., etc., etc. Now these are facts and not the fault of any clause of the constitution and by-laws. If we expect anything better, the presiding officer *must preside*, and to do so he *must* have help from the members.

Further you say: "Only one delegate votes, no matter how many go up from a given county society." Now I must insist that any delegation acting in this way plainly violates the constitution and by-laws of the association, for in Article VII, it expressly provides for *two* votes for *all* delegates *on all* questions, in adopting reports from nominating committees as well as every otherwise. Not only so, my dear " DANIEL'S MEDICAL JOURNAL," but if you will confer with a good lawyer and parliamentarian in regard to the force of this constitution and by-laws, poor as they are, you will be advised that this voting above referred to, is not a mere privilege to be exercised according to whim or individual notions, but a *duty* that every member and delegate ought to faithfully perform for the " greatest good to the greatest number." No ! the fault is carelessness and indifference of the members, and too much the case also with the officers. According to your view of the subject, taxation must be *only* on those who *are represented*, and not for a privilege or duty to be represented.

But notwithstanding, I want to vote " no good " for the constitution and by-laws of the Texas State Medical Association as it now stands under all the surrounding circumstances. As a member of committee on constitution and by-laws to report at our next session, I shall insist on doing away with all this double voting. It is too much trouble and too impracticable. Also let us have no more taxing local societies, except, perhaps, for some specific reason, or some *special* privilege.

And above all things else, I hope the association will adopt some plan of electing officers and committees by which we can have less demagogism and sneaking subterfuges in order to get into "place" than we have so unfortunately had in some cases in the last few years; men so losing, or else having so little dignity as to vote for themselves in order to be sure of office, which might do in politics, but in scientific bodies it smacks of littleness too much. Why not elect without nominations, and count the votes as in such cases in State, county and municipal elections in this State? *We* are *all* good fellows, and any man or woman good enough to be a member, *could* be good enough to fill *any* office for at least one term, at least long enough to be tried. Respectfully, etc.,

R. P. Talley, M. D.

